# Development of Focal Nodular Hyperplasia after Cyclophosphamide-Based Chemotherapy in a Patient with Breast Cancer

**DOI:** 10.1155/2018/5409316

**Published:** 2018-10-22

**Authors:** Dan-Qing Xue, Lan Yang

**Affiliations:** Department of Breast Surgery, The Third Affiliated Hospital of Suzhou University, 185 Juqian Road, 213003 Changzhou, China

## Abstract

Focal nodular hyperplasia (FNH) is the second most common liver cell-derived benign tumor. It is postulated that chemotherapy-induced hepatic circulatory abnormalities, like sinusoidal obstruction syndrome (SOS), could lead to the development of FNH. Cyclophosphamide was also reported to induce SOS in a synergistic effect with total body irradiation. However, none of cyclophosphamide-related FNH had ever been reported before. In this case report, we present a female patient who was treated with neoadjuvant chemotherapy of cyclophosphamide (500mg/m^2^)-docetaxel (75mg/m^2^)-pharmorubicin (90mg/m^2^) regimen every 3 weeks for breast cancer developed FNH after 4 courses of treatment. The patient had no chronic liver disease, no history of smoking, drinking, or medication use. The chronological correlation between the chemotherapy and the appearance of the FNH suggested a cause-effect association. Therefore, this is the first case report about development of FNH after cyclophosphamide-based chemotherapy. Taking into account the frequency of breast cancer, it is instructive to recognize such observation of FNH in the context to make the differential diagnosis with hepatic metastasis.

## 1. Introduction

Focal nodular hyperplasia (FNH) is the second most common liver cell-derived benign tumor [1]. The nature and pathogenesis of FNH remain controversial. It is postulated that chemotherapy-induced hepatic circulatory abnormalities, like sinusoidal obstruction syndrome (SOS), could lead to the development of FNH [2]. Alkylating agents, specifically busulfan and melphalan, was reported to be associated with the development of FNH in preparation for bone marrow transplantation due to the formation of SOS [3–5]. Cyclophosphamide, another kind of alkylating agents, was also reported to induce SOS in a synergistic effect with total body irradiation [4]. However, none of cyclophosphamide-related FNH had ever been reported before.

Neoadjuvant chemotherapy is increasingly used in breast cancer treatment to reduce the size of the primary tumor, eventually allowing radical or more conservative surgical intervention. The most commonly used neoadjuvant chemotherapeutic regimen is the combination of cyclophosphamide, anthracycline, and taxane derivatives. The patient in our case, who was treated with above chemotherapeutic agents for beast caner, developed FNH after 4 courses of treatment. This is the first case, to our knowledge, of development of FNH after cyclophosphamide-based chemotherapy.

## 2. Case Report

**Figure 1:**
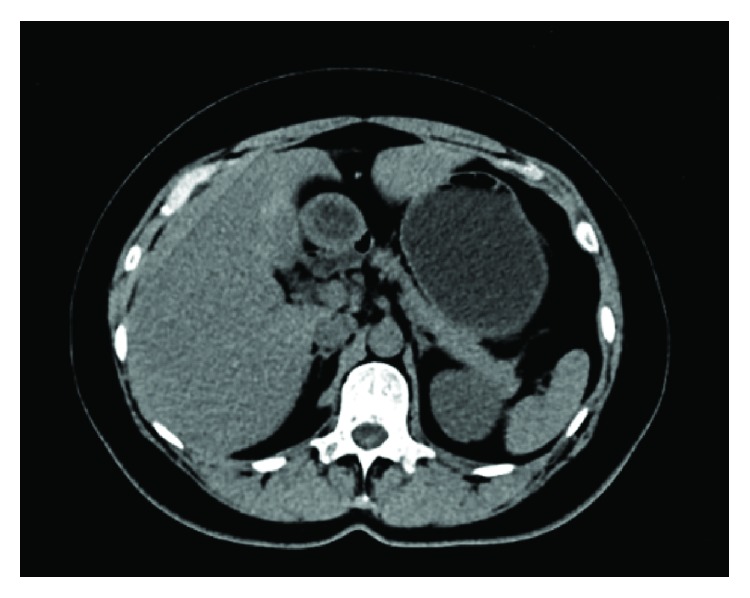
Computed tomography (CT) findings: plain CT scan performed at the diagnosis of cancer showed no hepatic abnormality.

**Figure 2:**
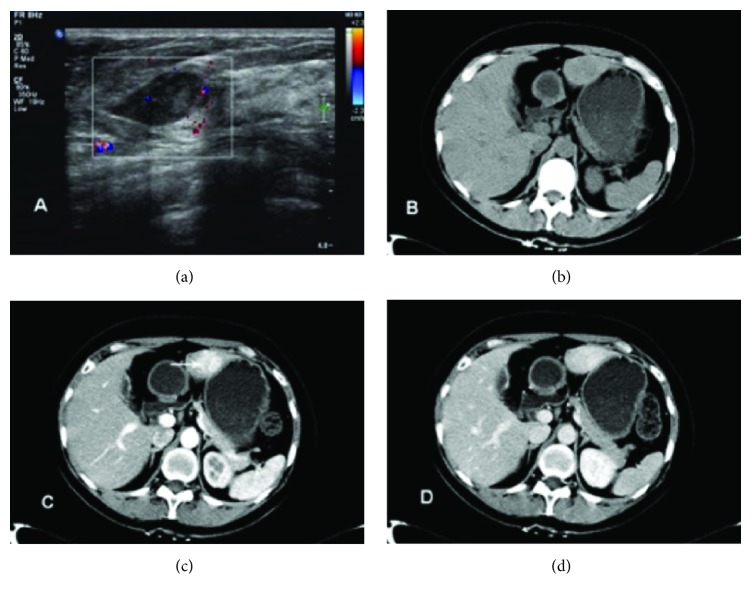
Abdominal ultrasonography (USG) and CT findings: (a) USG shows a 3 × 3cm slightly hypoisoechoic hepatic lesion in left lateral lobe with slightly unclear borders and blood flow signal; (b) plain CT shows that the lesion is lobulated, homogeneous, and isointense compared to surrounding liver parenchyma; (c) enhanced CT shows that hypervascular lesion appears hyperdense with a slightly hypodense central scar in the arterial phase (arrows); (d) in the portal venous phase the lesion is isodense without signs of central scar.

**Figure 3:**
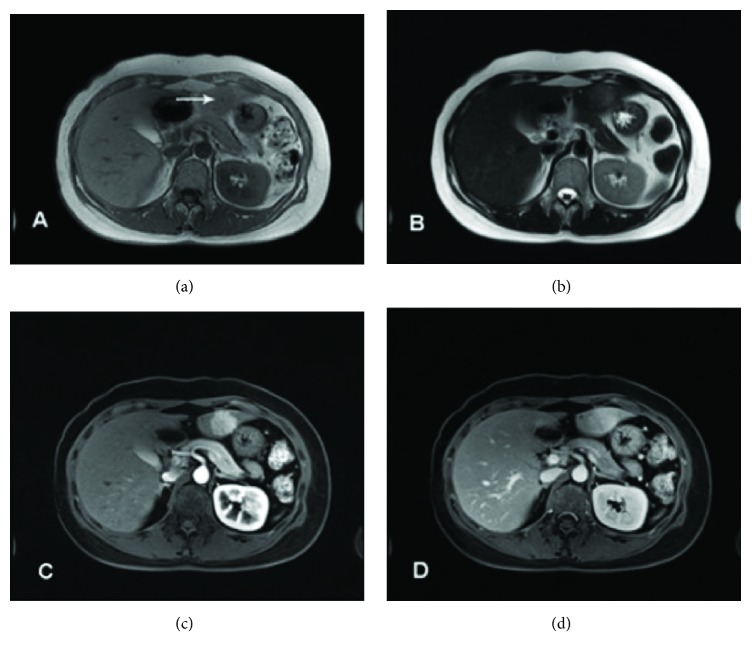
Abdominal magnetic resonance imaging (MRI) findings: (a) T1-weighted image shows that the lesion is slightly hypointense with the central scar appears more significant hypointense (arrow); (b) T2-weighted images shows the same lesion as slightly hyperintense; (c) after enhancement, the signal of the lesion is markedly increased and appears hyperintense during the arterial phase except for the central area in fat-saturated T1-weighted image; (d) in the portal venous phase, the lesion is slightly hyperintense compared to the normal liver tissue and the central scar is poorly recognized.

A 45-year-old woman was admitted to our breast surgery unit because of a palpable breast lesion in March 2014. After core biopsy she was diagnosed with breast cancer and received systemic neoadjuvant chemotherapy of cyclophosphamide (500mg/m^2^)-docetaxel (75mg/m^2^)-pharmorubicin (90mg/m^2^) regimen every 3 weeks. Computed tomography (CT) scan performed at the diagnosis of cancer and ultrasonography test before each cycle of chemotherapy showed no hepatic abnormality (Figure 1). After 4 courses of chemotherapy, a 3 × 3cm slightly hypoisoechoic hepatic lesion was identified in left lateral lobe on routine ultrasonography (USG) (Figure 2(a)). On subsequent plain CT scan, the lesion is homogeneous and isointense compared to surrounding liver parenchyma (Figure 2(b)). After enhancement, rapid homogeneous enhancement of lobular-shaped nodule was seen in the arterial phase with a slightly hypodense central scar (Figure 2(c)). The lesion is isodense without signs of central scar in the portal venous phase (Figure 2(d)). Abdominal magnetic resonance imaging (MRI) showed that the lesion is slightly hypointense on T1-weighted image with the central scar appears more significant hypointense (Figure 3(a)) and slightly hyperintense on T2-weighted images (Figure 3(b)). After enhancement, there is strong homogeneous enhancement of the lesion except for the central area during the arterial phase (Figure 3(c)) and slightly hyperintense in the portal venous phase (Figure 3(d)) compared to the normal liver tissue in fat-saturated T1-weighted image.

Both the patient's liver function serum values and tumor makers were within normal range and she had a negative hepatitis virus serology at the time when hepatic nodules were diagnosed. Besides, she had no history of smoking, drinking, or medication use and had no relevant family history. As the imaging findings could not be used to rule out the possibility of hepatocellular carcinoma, a histological examination was recommended. However, the patient refused to take liver biopsy due to personal reasons. Considering CT and MRI indicate the typical presentation of FNH, modified radical mastectomy was conducted afterwards and the patient was pathologically staged as pT2N2M0-GIII. The subsequent chemotherapy was 2 courses of the same regimen. The follow-up USG and CT showed that the lesion had been stable until the time of the report. She had been disease-free for more than 4 years.

## 3. Discussion

The case we present suggested that the development of FNH is potentially induced by chemotherapy. The patient had no chronic liver disease, no history of smoking, drinking, or medication use. The chronological correlation between the chemotherapy and the appearance of the FNH suggested a cause-effect association.

FNH, a hyperplastic nodule that contains scar-like tissue, is the second most common liver cell-derived benign tumor [1]. Until now, the nature and pathogenesis of FNH remain unclear. It is postulated that hepatic circulatory abnormalities including portal venous thrombosis, vascular recanalization, and reperfusion could lead to the development of FNH after chemotherapy [2]. SOS is kind of chemotherapy-induced damage to sinusoidal integrity, which could cause circulatory abnormalities [6]. Oxaliplatin-based systemic chemotherapy for the patients with colorectal liver metastasis was mostly reported to be associated with the development of FNH due to the formation of SOS [7–10]. According to previous researches, alkylating agents, specifically busulfan and melphalan, are associated with the development of FNH when administered prior to bone marrow transplant due to SOS [7]. It is also reported that cyclophosphamide is attributed to induce SOS in a synergistic effect with total body irradiation [4]. The potential mechanism might be depletion of reduced glutathione by cyclophosphamide in sinusoidal endothelial cells which make themselves more vulnerable to irradiation hazard [3–5]. In our case, the patient received standard cyclophosphamide-docetaxel-pharmorubicin regimen. The combined chemotherapy makes it difficult to conclude the specific pathogenic agent. Because there is no literature which reported the association among FNH, docetaxel, and pharmorubicin, cyclophosphamide seemed to be the most possible causative factors.

Because most FNH are asymptomatic and are without carcinomatous change, nonoperative therapy is the standard treatment. However, it is important to make accurate diagnosis of patient who develop hepatic nodule during chemotherapy because FNH and metastatic disease of the liver have total different prognoses. MRI is thought to be the best noninvasive method for diagnosing FNH at present whose sensitivity and specificity are 70% and 98%, respectively [11]. It is easy to make a definitive diagnosis of classical FNH when MRI indicates that tumor quality is homogeneous with a central scar and there is hypervascularity with enhancement during the arterial phase [12]. Otherwise, aspiration biopsy is required when it is difficult to obtain a definitive diagnosis only by imaging techniques.

In our case, we diagnosed FNH by combining imaging examinations including USG, CT, and MRI, by which the rate of definite diagnosis was reported up to 90% [13]. However, as hepatocellular carcinoma and metastatic renal cell carcinoma were reported to locate within the confines of FNH [14, 15], a certain period of follow-up time is required when nonoperative therapy is conducted. In this case, the patient had been asymptomatic for more than 4 years and the FNH was stable in size until the time of the report.

## 4. Conclusion

This is the first case report about development of FNH after cyclophosphamide-based chemotherapy. It could help confirm the damage effect of cyclophosphamide on hepatic sinusoidal integrity and may provoke further investigation on this area. On the other side, as increasing proportion of breast cancer patients will receive neoadjuvant chemotherapy, we should keep the possibility under consideration if new liver lesion mimicking hepatic metastasis was detected after cyclophosphamide-based chemotherapy in clinical practice.

## References

[1] Bonney G. K., Gomez D., Al-Mukhtar A., Toogood G. J., Lodge J. P. A., Prasad K. R. (2007). Indication for treatment and long-term outcome of focal nodular hyperplasia. *HPB*.

[2] Joyner B. L., Goyal R. K., Newman B., Levin T. L. (2005). Focal nodular hyperplasia of the liver: a sequela of tumor therapy. *Pediatric Radiology*.

[3] McDonald G. B., Slattery J. T., Bouvier M. E. (2003). Cyclophosphamide metabolism, liver toxicity, and mortality following hematopoietic stem cell transplantation. *Blood*.

[4] Holter-Chakrabarty J. L., Pierson N., Zhang M. J., etal. (2015). The Sequence of Cyclophosphamide and Myeloablative Total Body Irradiation in Hematopoietic Cell Transplantation for Patients with Acute Leukemia. *Biol Blood Marrow Transplant*.

[5] Antman K., Eder J. P., Elias A. (1987). High-dose combination alkylating agent preparative regimen with autologous bone marrow support: The Dana-Farber Cancer Institute/Beth Israel Hospital experience. *Cancer Treatment Reports*.

[6] Fan C. Q., Crawford J. M. (2014). Sinusoidal obstruction syndrome (hepatic veno-occlusive disease). *Journal of Clinical and Experimental Hepatology*.

[7] Icher-De Bouyn C., Leclere J., Raimondo G. (2003). Hepatic focal nodular hyperplasia in children previously treated for a solid tumor: Incidence, risk factors, and outcome. *Cancer*.

[8] Brisse H., Servois V., Bouche B. (2000). Hepatic regenerating nodules: A mimic of recurrent cancer in children. *Pediatric Radiology*.

[9] Kumagai H., Masuda T., Oikawa H., Endo K., Endo M., Takano T. (2000). Focal nodular hyperplasia of the liver: Direct evidence of circulatory disturbances. *Journal of Gastroenterology and Hepatology*.

[10] Rubbia-Brandt L., Audard V., Sartoretti P. (2004). Severe hepatic sinusoidal obstruction associated with oxaliplatin-based chemotherapy in patients with metastatic colorectal cancer. *Annals of Oncology*.

[11] Li A.-J., Zhou W.-P., Wu M.-C. (2006). Diagnosis and treatment of hepatic focal nodular hyperplasia: report of 114 cases. *Chinese Journal of Surgery*.

[12] Terkivatan T., Van Den Bos I. C., Hussain S. M., Wielopolski P. A., De Man R. A., Ijzermans J. N. M. (2006). Focal nodular hyperplasia: Lesion characteristics on state-of-the-art MRI including dynamic gadolinium-enhanced and superparamagnetic iron-oxide-uptake sequences in a prospective study. *Journal of Magnetic Resonance Imaging*.

[13] Shen Y. H., Fan J., Wu Z. Q., etal. (2007). Focal nodular hyperplasia of the liver in 86 patients. *Hepatobiliary & Pancreatic Diseases International*.

[14] Langrehr J. M., Pfitzmann R., Hermann M. (2006). Hepatocellular carcinoma in association with hepatic focal nodular hyperplasia. *Acta Radiologica*.

[15] Wheeler Y. Y., Wheeler G. L., Diaz-Arias A. A., Anders R. A. (2009). Metastatic renal cell carcinoma within a hepatic focal nodular hyperplasia: A case report and review of the literature. *International Journal of Clinical and Experimental Pathology*.

